# Hydration and Microstructure of Cement Pastes with Calcined Hwangtoh Clay

**DOI:** 10.3390/ma12030458

**Published:** 2019-02-01

**Authors:** Run-Sheng Lin, Xiao-Yong Wang, Han-Seung Lee, Hyeong-Kyu Cho

**Affiliations:** 1 Department of Architectural Engineering, Kangwon National University, Chuncheon-Si 24341, Korea; linrunsheng@kangwon.ac.kr; 2 Department of architectural engineering, Hanyang University, Ansan-Si 15588, Korea; ercleehs@hanyang.ac.kr; 3 Energy and environment division, Korean Institute of Ceramic Engineering and Technology, Jinju-Si 52851, Korea; hkcho@kicet.re.kr

**Keywords:** water-to-binder ratio, composite binder, calcined Hwangtoh clay, hydration

## Abstract

Calcined Hwangtoh (HT) clay is a very promising supplementary cementitious material (SCM). In this work, the development of the mechanical properties and microstructures of HT-blended cement paste was studied after substituting the binder with HT powder calcined at 800 °C. The water-to-binder (w/b) ratios of the paste used were 0.2 and 0.5, and the quantities of HT powder added to the mixture were 0, 10, and 20%. The compressive strength test indicates that the addition of the HT powder increases the compressive strength of the paste after seven days of curing, and the highest compressive strength is obtained with the 10% HT substitution, regardless of whether the w/b ratio is 0.5 or 0.2. X-ray fluorescence (XRF), X-ray diffraction (XRD), inductively coupled plasma mass spectrometry (ICP-MS), isothermal calorimetry, thermogravimetric analysis (TGA), and attenuated total reflection Fourier transform infrared spectroscopy (ATR-FTIR) analysis show that the HT powder not only has a physical effect (i.e., nucleation effect and dilution effect) on cement hydration but also has a chemical effect (i.e., chemical reaction of HT). The results of scanning electron microscopy (SEM) and mercury intrusion porosimetry (MIP) analysis show that the paste has more ettringite during the early stage, and the microstructure is refined after the addition of the HT powder. In addition, the relationships between chemically bound water, hydration heat, and compressive strength are presented.

## 1. Introduction

Cement is currently the most manufactured product on earth and has made tremendous contributions to human development. However, the production of cement has also created a significant environmental pollution problem. According to a report by Malhotra [1], the annual global greenhouse gas (GHG) emissions produced by ordinary Portland cement (OPC) are estimated to be close to 1.35 billion tons, equivalent to about 7% of total GHG emissions. Roughly estimated data indicate that one ton of Portland cement can release about one ton of carbon dioxide (CO_2_) into the air [2]. Therefore, in order to reduce the amount of cement used, supplementary cementitious materials (SCMs) are attracting increasing attention. Golewski divided SCMs into six categories: industrial wastes, nano-industrial wastes, agricultural wastes, aquaculture wastes, minerals, and dust and powders [3]. There is a global increase in the use of SCMs, such as fly ash, ground granulated blast furnace slag, silica fume, and metakaolin. This is because concrete mixtures can be rendered more economical and environmentally friendly by using SCMs, which can reduce the permeability, increase the strength, or improve other properties of concrete through filler effects and pozzolanic reactions [4,5,6].

Meanwhile, with the aim of finding solutions to the environmental pollution caused by cement, the Environment Program Sustainable Building and Climate Initiative (UNEP-SBCI) presented the main conclusions of a low-CO_2_, eco-efficient cement-based materials analysis [7]. The report mentions that the partial replacement of clinker with calcined clay could effectively reduce the environmental pollution caused by the manufacture of cement. According to the data provided by Long [8], the global warming potential (GWP) of calcined clay is 0.4 CO_2_ kg/kg, which is much lower than the GWP of cement (0.83 CO_2_ kg/kg). It is well known that clays, especially those containing kaolinite, produce active substances when calcined to about 600–900 °C [9,10]. It is understood that calcined clay can be produced in equipment similar to that used for Portland cement with similar investment costs, but the calcination temperature of clay is much lower than that of clinker (1450 °C), resulting in lower energy costs [11]. Moreover, the reserves of clay are so large that they are virtually infinite compared with the amount of cement produced.

Hwangtoh clay (HT) is a unique clay in Korea and is mainly produced by the weathering of rocks [12]. The storage of HT is sufficiently abundant to ensure its capacity for large-scale industrial use. HT has many eco-environmental advantages, including purification, deodorization, and disinfection. HT is also used as a cosmetic material in some aesthetic and medical institutions in Korea. The environmental and health benefits of HT are unmatched by other SCMs. Due to the various advantages of HT, it has received widespread attention in recent years, especially in the field of building materials. For example, Koo et al. [13] placed mice in a mortar cage made of calcined HT and compared them with mice kept in an OPC mortar cage. The activity parameters of the mice (i.e., weight, food intake, water intake, reproductive activity, and aggression) fed in the calcined HT environment were found to be better than those of the mice in the OPC mortar cage. Moreover, the results of another study by Koo et al. [14] showed that the compressive strength, elastic modulus, and pH of HT-concrete are similar to those of ordinary cement concrete. Go et al. [15] showed that the characteristics of HT are closely related to the calcination temperature. In their study [15], when HT was calcined at 800 °C and used as a substitute for part of the cement clinker, the mechanical properties were significantly improved compared with the OPC samples. Uncalcined HT is similar to kaolin, and it requires high-temperature calcination to achieve pozzolanic activity [12,13,16,17]. The main phases of uncalcined HT are illite, kaolinite-montmorillonite, quartz, and a small amount of triclinic feldspar. However, after calcination at a high temperature, kaolinite-montmorillonite disappears, and calcined HT displays pozzolanic activity [15].

Although it is known to be an environmentally friendly and health beneficial material, HT has not been fully explored for its potential application in concrete production. Of particular interest are the hydration and microstructure of HT-blended cement with a relatively low water-to-binder (w/b) ratio (0.2), which is typical of ultra-high-performance concrete (UHPC). To successfully use HT in the concrete industry, it is necessary to perform a systematic study on the physical and chemical effects of HT on the hydration and microstructure of the resulting material. For this thesis, the HT powder substitution ratios are 10 and 20%. For paste mixture samples, the w/b ratios are 0.5 and 0.2. The effects of the HT powder on cement hydration performance were analyzed using a compressive strength test, X-ray fluorescence (XRF) spectroscopy, X-ray diffraction (XRD), inductively coupled plasma mass spectrometry (ICP-MS), scanning electron microscopy (SEM), attenuated total reflection Fourier transform infrared spectroscopy (ATR-FTIR), thermogravimetric analysis (TGA), mercury intrusion porosimetry (MIP), and isothermal calorimetry. This study will provide guidance for future research that involves the addition of calcined HT to ordinary concrete and UHPC.

## 2. Experimental Procedure

### 2.1. Materials

**Figure 1:**
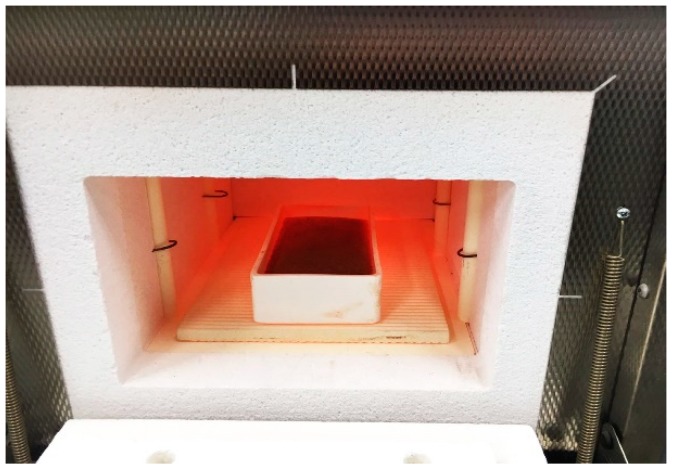
Calcination process of Hwangtoh powder.

Uncalcined HT clay was obtained from the Jeolla-Do area of South Korea (Insan-Si). Calcined HT was produced according to the high-temperature calcination method reported by Go et al. [15]. The HT clay was calcined at 800 °C for one hour using a muffle furnace, as shown in Figure 1, then rapidly cooled in air.

**Figure 2:**
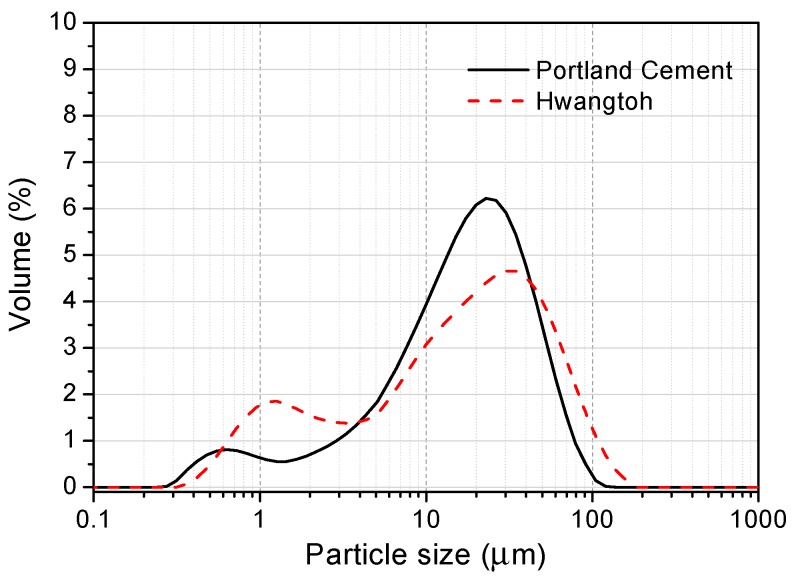
The distributions of the particle sizes of the tested materials.

As reported in Table 1, the chemical compositions of HT clay and ordinary Portland cement (Type 1) were analyzed using X-ray fluorescence (ZSX Primus II, Rigaku, Tokyo, Japan) spectrometry. The total content of Al and Si elements in HT powder is close to 90%, which is much higher than that in OPC. The particle size distribution (PSD) analysis indicates that the average particle sizes of HT and OPC are 12.1 and 18.6 μm, respectively (Figure 2). The specific gravity of the cement and Hwangtoh powder was tested according to ASTM C188 and is 3.14 and 2.8, respectively.

### 2.2. Mix Design Information

The design information for the mix ratios can be seen in Table 2. The six paste samples are denoted by 02HT00, 02HT10, 02HT20, 05HT00, 05HT10, and 05HT20. The HT powder was used to replace 10 and 20% of the total weight of the binder, and the resulting mixtures were compared with the control sample (containing only Portland cement clinker). All the pastes were tested for flowability using a mini-slump flow test [18], and the flow values are shown in Table 2. The aluminum phase in HT powder is known to cause an increase in the amount of superplasticizer [19,20]. Also, the particle size analysis (Figure 2) reveals that the HT powder particles are finer than the cement particles; this causes a decrease in the mixture’s flowability after the addition of HT. Therefore, to obtain similar flowabilities, superplasticizers were added at 0.6, 1.0, and 1.4% of the binder mass to the 02HT00, 02HT10, and 02HT20 mixtures, respectively. In addition, the finer particles of the HT powder can reduce the bleeding of the paste after the addition of HT.

### 2.3. Methods

The mixtures were introduced into a mechanical agitator, mixed, and poured into a metal mold with dimensions 40 mm × 40 mm × 160 mm. They were then coated with plastic film. One day later, the mold was dismantled, and the samples were wrapped with plastic film. Afterward, the samples were placed in a chamber at 20 °C until testing the compressive strength of the samples after 3, 7, and 28 days in accordance with ASTM C349.

The XRF data show that the HT powder contains a large proportion of aluminum. In order to measure the dissolution of aluminum ions (Al^3+^), the Al^3+^ concentrations in mixtures with different HT amounts were measured by inductively coupled plasma mass spectrometry (ICP-MS) (NexION 300D, PerkinElmer, Waltham, MA, USA). Ten grams of solid powder (HT powder accounted for 0, 10, and 20% of the solid mass, respectively) was mixed with 100 g of deionized water and stirred well for one hour with a magnetic stirrer. The upper layer of the liquid was obtained using a vacuum suction device after being sealed for one day in a chamber at 20 °C. The concentration of the Al^3+^ ions in the three groups of liquids were then measured separately using ICP-MS [21].

Microscopic observations of the samples were made using high-resolution field emission scanning electron microscopy (S-4300, Hitachi, Tokyo, Japan). The surface of the sample was coated with platinum using an E-1010 ion sputter from Hitachi [22]. Each sample was measured at a curing age of three days.

The heat release rate and accumulated hydration heat of the adhesives were measured using a TAM-Air (TA Instruments, New Castle, DE, USA) for 72 h at 20 °C. Because the temperature can differ between the paste and the calorimeter’s internal environment, the binder and water temperatures were kept as close to 20 °C as possible before mixing. After mixing using a mechanical agitator, 10 g of the paste was removed and added to an ampoule. This was then put it in the calorimeter as quickly as possible [23].

The XRD analysis was performed using a PANalytical X’pert Pro MPD diffractometer (PANalytical, Almelo, the Netherlands) with Cu Kα radiation (λ = 1.5408 Å). The samples were scanned from 5 to 80° (2θ) in steps of 0.013° (2θ), with a cumulative time of 8.67 s per step [22].

The TGA data were acquired using the LABSYS EVO Series (Setaram, Caluire, France). The heating rate was 10 °C/min, and the temperature range was between room temperature and 1050 °C. Nitrogen was continuously supplied throughout the test. The contents of chemically bound water and calcium hydroxide were calculated at 105–1050 °C and 400–550 °C, respectively. Since unreacted cement and HT have a loss on ignition, the chemically bound water content and calcium hydroxide content must be corrected by the loss on ignition of the unreacted mixture [24].

Infrared spectroscopy can reflect changes in the vibrational energy of the molecule, and qualitative analysis of the substances in the pastes can be performed according to the position and shape of the absorption peak. ATR-FTIR spectra of the specimens were recorded using a Frontier spectrometer (PerkinElmer, Waltham, MA, USA) in the range between 4000 and 500 cm^−1^, with a 0.4 cm^−1^ resolution and 32 scans for each sample [25]. Each sample was measured at a curing age of 28 days.

The Autopore IV Series Automatic Mercury Porosimeter (Micromeritics, Norcross, GA, USA) was used for the MIP analysis. The instrument consists of four low-voltage systems and two high-voltage systems. During the test, the samples were put into a dilatometer (sample container), which was then loaded into the mercury porosimeter for testing [26].

## 3. Results and Discussion

### 3.1. Characterization of the Calcined HT

**Figure 3:**
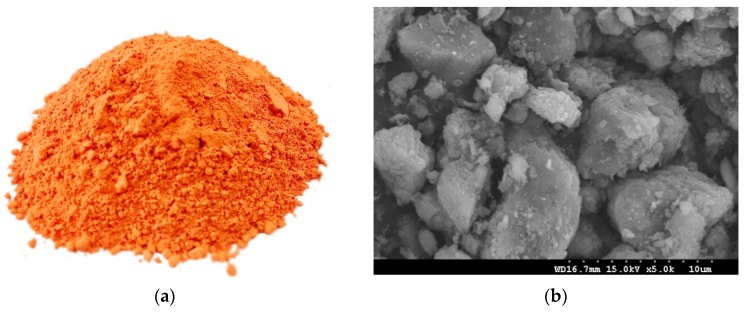
(**a**) Calcined Hwangtoh powder. (**b**) SEM micrographs of calcined Hwangtoh.

**Figure 4:**
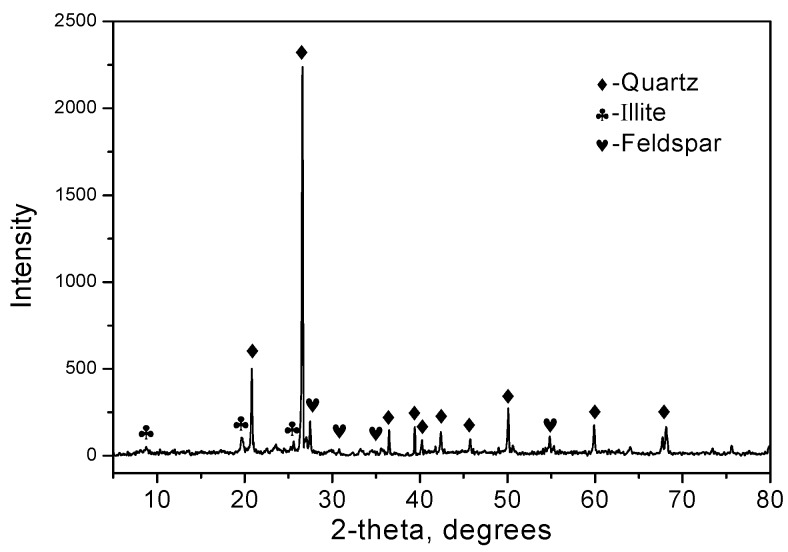
XRD patterns of calcined Hwangtoh powders.

An HT powder specimen and the SEM micrographs of the calcined Hwangtoh powder are presented in Figure 3. The HT particles are irregular in shape and have a rough surface. The size of the particles is between approximately 1 and 15 μm, which is similar to the results of the PSD analysis (Figure 2). A small particle size can provide more nucleation sites during cement hydration and promote the early hydration of cement [27,28]. The XRD patterns of the calcined Hwangtoh powder (Figure 4) show that the kaolinite-montmorillonite peak (2θ = 12.36°) essentially disappears after calcination, which is consistent with the phenomenon observed by Go et al. [15]. This clearly shows that, after the heat treatment, phase transformation of HT occurred. Quartz, feldspar, and a small amount of illite are also evident in the XRD pattern. The observed feldspar appears to be a triclinic-type feldspar, usually considered microcline.

### 3.2. Compressive Strength

HT powders influence cement hydration by three major mechanisms: nucleation, dilution, and chemical action. HT particles can serve as nucleation sites during the hydration of cement and accelerate the hydration process. This is known as the nucleation effect [17,29,30], and it has a positive influence on the strength of the mix. When HT replaces partial cement, the content of cement is reduced, which is the dilution effect. HT’s dilution effect on cement can increase the effective water-to-cement (w/c) ratio, thus accelerating the hydration of cement and improving the compressive strength of the mix [31]. However, it also leads to a reduction in the cement content of the paste, which has a negative effect on the strength of the mix [27,31]. The chemical effect of HT on the paste during the early stage of the curing is mainly due to the aluminum phase; the silicon phase primarily affects the later stage and also has an enhancing effect on the strength [32].

**Figure 5:**
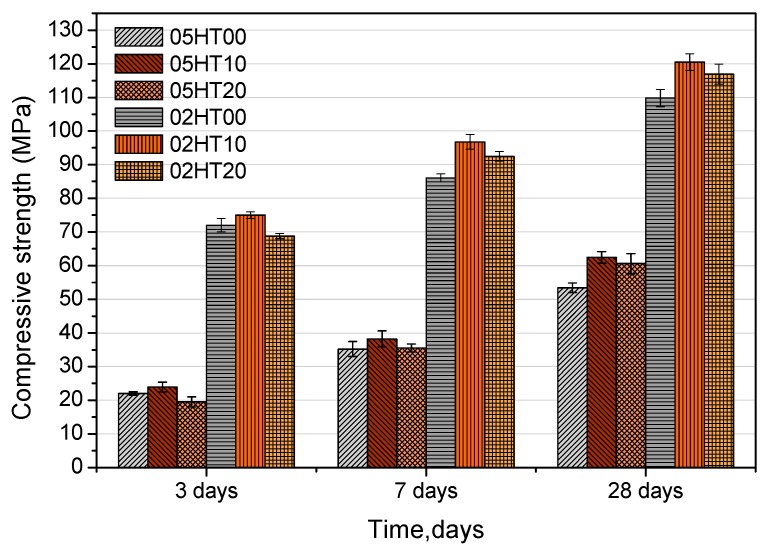
Comparison of compressive strength of samples with different Hwangtoh powder contents.

Figure 5 shows the compressive strength of the samples with w/b ratios of 0.5 and 0.2. On day 3, regardless of the w/b ratio, the samples with 10% HT have a slightly increased compressive strength. However, for the samples containing 20% HT powder, the compressive strength is reduced by 2.5 MPa compared with the control. There is a great probability that, compared with cement clinker, the early activity of the HT powder is lower. After seven days, the HT-blended cement paste with a w/b ratio of 0.2 has a higher strength. Compared with the control, the strength of the 02HT10 sample is increased by 10.5 MPa, while that of the 02HT20 sample is increased by 6.4 MPa. However, the strength of the samples with a w/b value of 0.5 does not change significantly, and the 05HT10 sample increases only slightly compared with the control. This is because the dilution effect on the strength is more significant when there is a low water-to-cement ratio. At 28 days, there is a significant improvement in the strength of all the HT-blended cement pastes. In particular, compared with the control, 05HT10 and 02HT10 are increased by 9.02 and 10.7 MPa, respectively. This may be due to the contribution of the silicon phase in HT to the compressive strength.

### 3.3. Hydration Heat

**Figure 6:**
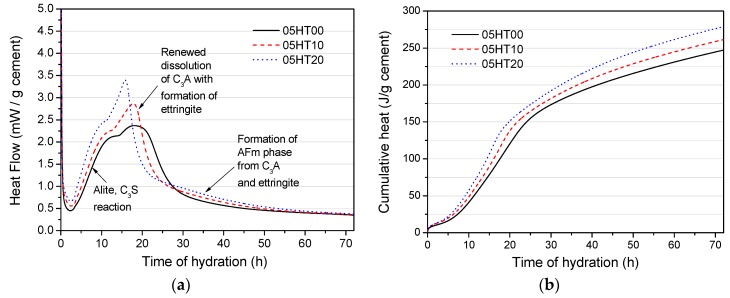
Hydration heat evolution for samples with w/b = 0.5. (**a**) Heat flow and (**b**) cumulative heat.

The heat flow and the accumulated heat of the pastes with a w/b ratio of 0.5 are shown in Figure 6. The results are normalized per gram of OPC. From Figure 6a, it is obvious that the main exothermic peak of the hydration rate is enhanced by the addition of the HT powder. Gutteridge and Dalziel [33] first determined that this phenomenon is due to the filler surface providing extra nucleation sites for calcium silicate hydrate (C-S-H). During the acceleration of the reaction rate, alite reacts rapidly, at which time the C-S-H and the Portlandite grow rapidly [34]. During the deceleration period of the main exothermic peak, the reaction of the aluminate phase results in the rapid formation of ettringite [6]. At the later stage of the deceleration period, calcium monosulfoaluminate (AFm) gradually forms [35]. In Figure 6b, the accumulated heats are normalized per gram of OPC. The accumulated heat values of samples with HT powder added are increased remarkably, and the value increases as the replacement rate increases. For the pastes of 05HT10 and 05HT20, the heat values increase by 14.24 and 31.76 J/g, respectively, compared with the control.

**Figure 7:**
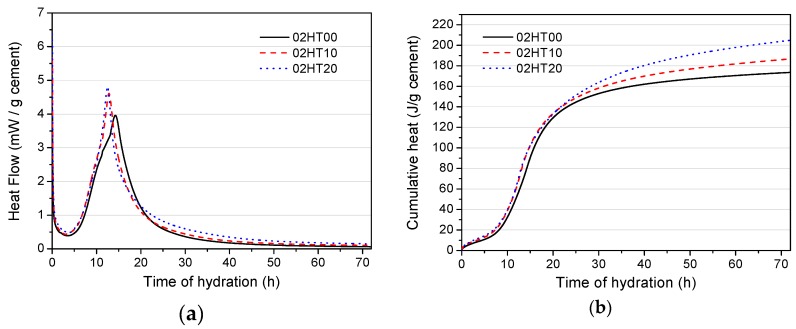
Hydration heat evolution of samples with w/b = 0.2. (**a**) Heat evolution rate. (**b**) Cumulative heat.

Figure 7 shows the heat evolution rate and the cumulative hydration heat for w/b = 0.2. From Figure 7a, it can be clearly seen that when w/b = 0.2, similar to when w/b = 0.5, the addition of HT promotes the hydration heat rate of OPC. However, it is worth mentioning that, compared with Figure 6a, the main exothermic peak of the low w/b ratio is significantly steeper, and the maximum exothermic rate is raised. This experimental phenomenon is the same as that found by Hu et al. [36]. One possible reason is that a lower water-to-cement ratio results in higher ion concentrations, which may lead to higher hydration rates and accelerated early hydration [37].

Generally, superplasticizers delay the initial hydration of the cement [38]. However, it can be seen from Figure 7a that, although the HT-added pastes were doped with more superplasticizers, there is no delay in the hydration of the cement. This may be due to the aluminum phase in the HT powder adsorbing a part of the superplasticizer [19,20]. At the same time, as discussed above, HT can accelerate the progress of cement hydration. This also offsets the delayed effects of the superplasticizer. Figure 7b reports the test results according to the standard per gram of OPC. The addition of HT increases the accumulated heat value. Compared with the control group, 02HT10 is increased by 13.14 J/g, while 02HT20 is increased by 31.32 J/g.

**Figure 8:**
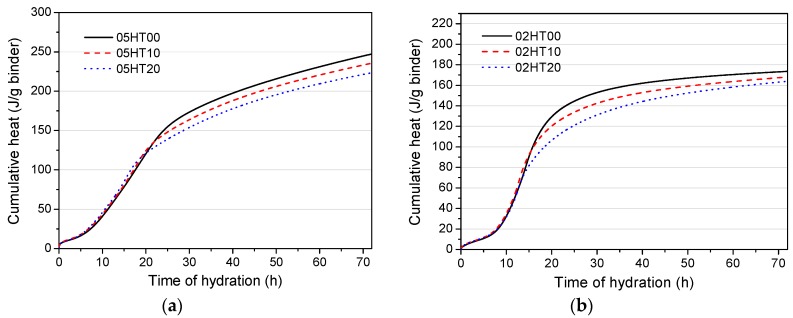
Cumulative heat of binder pastes normalized per gram of binder when (**a**) w/b = 0.5 and (**b**) w/b = 0.2.

As can be seen from Figure 8, the results of the accumulated heat exotherms are normalized per gram of binder. It is apparent that the HT-containing paste has a lower total heat release compared with the pure cement paste. The cumulative heat of 05HT20 and 02HT20 is 9.63 and 5.76% lower than that of the control group, respectively. The accumulated hydration heat value of samples with a w/b of 0.5 is higher than those with w/b = 0.2. Moreover, the trend of the hydration heat continues to rise when w/b = 0.5, while the trend tends to be flat when w/b = 0.2.

### 3.4. Aluminum Ion Concentration

The basic feature of all silicate structures is the structural unit ${\mathrm{SiO}}_{4}^{4-}$. Four O^2−^ groups are arranged in a regular tetrahedron around Si^4+^ to form a silica tetrahedron. Since the cation can be replaced by other similarly sized cations via isomorphous substitution, the Al^3+^ substitution of Si^4+^ is completely arbitrary and indefinite, forming an aluminosilicate [39,40]. Table 3 shows the concentration of the Al^3+^ ions dissolved in the supernatant. After one day, the Al^3+^ ion concentrations for 100% OPC, 90% OPC + 10% HT, and 80% OPC + 20% HT are 2.24, 2.69, and 3.36 μg/L, respectively. As the amount of HT added increases, the concentration of Al^3+^ ions also increase. This means that the aluminum phase in HT can release more Al^3+^ ions in the early stage than OPC. This also explains why pastes with HT powder form more pronounced peaks of C_3_A: renewed dissolution with the formation of ettringite (Figure 6) in the early stage of hydration heating.

### 3.5. Paste Microstructure

**Figure 9:**
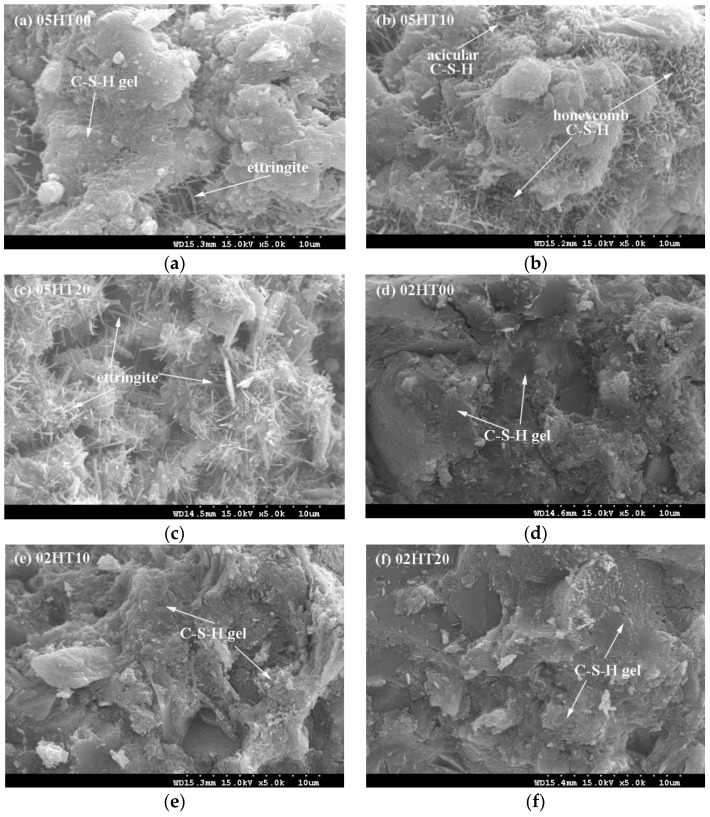
Microstructure images of three-day old pastes. (**a**) 05HT00, (**b**) 05HT10, (**c**) 05HT20, (**d**) 02HT00, (**e**) 02HT10, and (**f**) 02HT20.

Figure 9 shows SEM micrographs of the pastes after three days. From Figure 9a–c, the 05HT20 paste has more acicular structures of ettringite than the control. The Al^3+^ ion concentration test results of ICP-MS show that the addition of 20% HT powder significantly increases the concentration of Al^3+^ ions. Since the formation of ettringite is closely related to the aluminum phase, it can be confirmed that the aluminum phase in the HT powder participates in hydration. The results from the heat of hydration (Figure 6a) also demonstrate that the addition of HT powder promotes the formation of ettringite. In Figure 9b, large amounts of acicular C-S-H and honeycomb C-S-H appear on the surface of the particles. The hydration product of C-S-H first grows in a diffuse manner, then densifies [41]. As can be seen from Figure 9d–f, all the pastes produce dense C-S-H hydration products at low w/b ratios. Additionally, the HT powder is well fused with the hydration product at the three-day curing age. Moreover, the acicular structures of ettringite are not observed in the microscopic images of samples with w/b = 0.2 because of the insufficient space and capillary water.

### 3.6. Thermogravimetric Analysis

**Figure 10:**
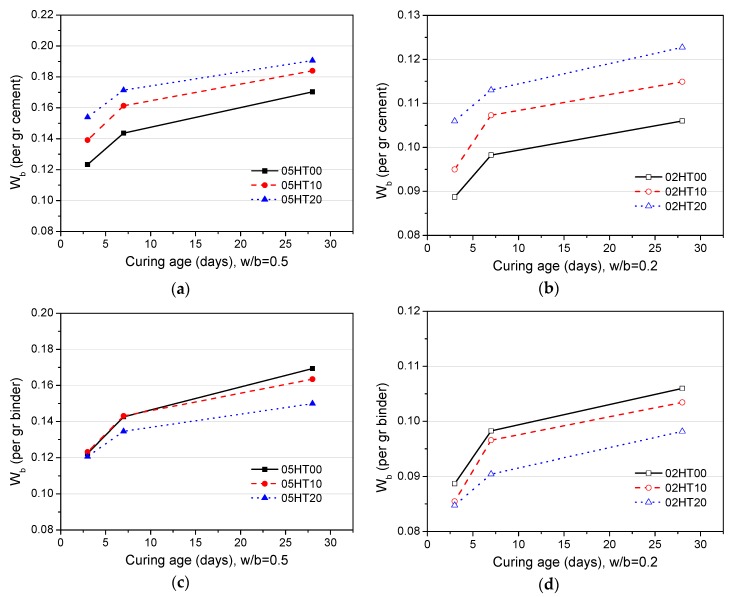
W_b_ content per gram of cement when (**a**) w/b = 0.5 and (**b**) w/b = 0.2. W_b_ content per gram of binder when (**c**) w/b = 0.5 and (**d**) w/b = 0.2.

Figure 10 shows the chemically bound water (W_b_) content in pastes with a w/b of 0.5 and 0.2. Additionally, Figure 10a,b show the results normalized per gram of OPC. As discussed above, HT has physical and chemical effects on the hydration of cement. Therefore, regardless of the w/b ratio, the W_b_ of the HT-blended cement pastes are higher than that of the control. This is due to the filler effect of the HT powder and the reaction of the aluminum and the silicon phases in HT. After 28 days, the W_b_ content of 05HT20 is increased by 11.8% compared with the 05HT00 paste (Figure 10a), and the W_b_ content of 02HT20 is increased by 15.8% compared with the 02HT00 paste (Figure 10b). Compared with a w/b ratio of 0.5, the paste with the 0.2 w/b ratio increases more significantly. One of the reasons is that, when the water-to-cement ratios are lower, the dilution effect is more pronounced [24,27]. Figure 10c,d show results that are normalized per gram of binder. As the replacement ratio of HT increases, W_b_ decreases. When the value of w/b decreases from 0.5 to 0.2, W_b_ also decreases.

**Figure 11:**
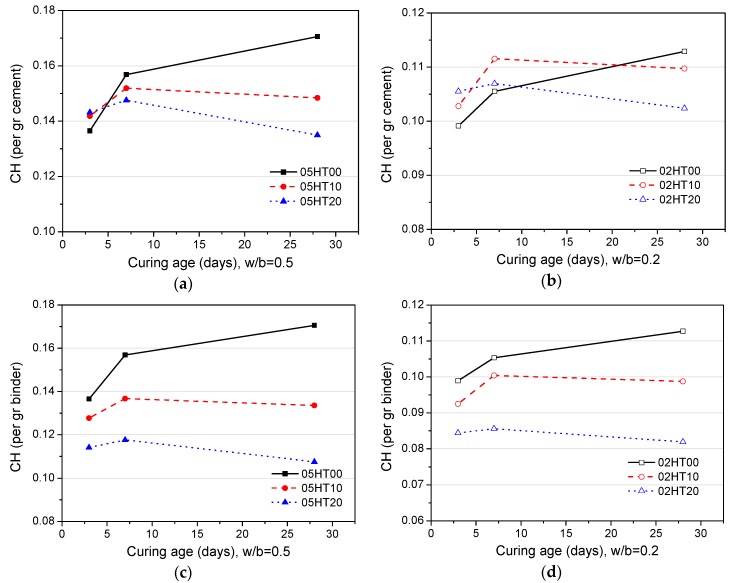
Calcium hydroxide (CH) content per gram ofordinary Portland cement (OPC) when (**a**) w/b = 0.5 and (**b**) w/b = 0.2. CH per gram of binder when (**c**) w/b = 0.5 and (**d**) w/b = 0.2.

Figure 11 shows the content of calcium hydroxide (CH) in the pastes. Figure 11a,b show the results normalized per gram of OPC. The content of CH depends on the CH generated by OPC hydration and the CH consumed by the reaction with the HT powder [13,16,42]. At the three-day curing age, the CH content is higher than that of the control, regardless of whether the w/b ratio is 0.5 (Figure 11a) or 0.2 (Figure 11b). This is because the filler effect of the HT powder can promote the early hydration of cement and, thus, generate more CH. That is, the content of CH produced is greater than that consumed. After seven days, the CH content of the HT-added pastes with a w/b ratio of 0.5 is less than that of the OPC pastes. This is because the silicon phase in HT starts to react with CH and consumes a large amount of CH. However, in the pastes with a w/b ratio of 0.2, the CH content of the HT-added pastes is still higher than that of the control. This is because the dilution effect of HT is more prominent for a lower w/b ratio, and there is a considerable increase in cement hydration, resulting in the production of more CH. At the age of 28 days, the CH content of the HT-blended cement pastes with w/b ratios of 0.5 and 0.2 are significantly lower than that of the control. Figure 11c,d show results that are normalized per gram of binder. As the replacement ratio of HT increases, the CH content decreases. As the w/b ratio decreases from 0.5 to 0.2, CH also decreases.

### 3.7. X-Ray Powder Diffraction

**Figure 12:**
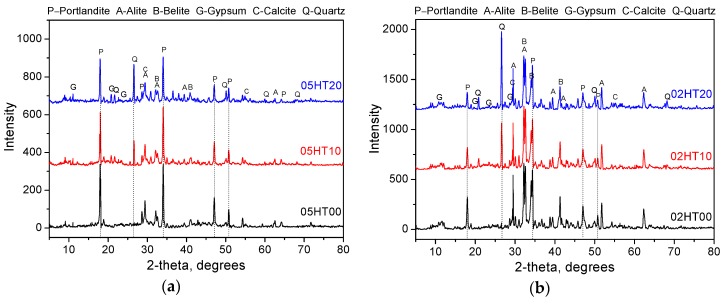
X-ray diffraction curves obtained for all the samples at 28 days when (**a**) w/b = 0.5 and (**b**) w/b = 0.2.

Figure 12 shows the XRD patterns of all samples after 28 days. From the results of XRD analysis, it can be seen that portlandite, alite, belite, calcite, gypsum, and quartz are the main crystal phases. The quartz phase is a crystal phase unique to HT powder. The presence of the calcite phase may be due to the carbonization during the experiment. It can be seen from the diffraction pattern that the intensity of the portlandite peak (main peaks at 18.08, 28.66, 34.08, and 47.12°, 2θ) decreases as the amount of HT added increases, regardless of the w/b ratio. This is consistent with the thermogravimetric analysis. In samples with a w/b of 0.2, the peak intensities of alite and belite are significantly higher than the peak of samples with a w/b of 0.5 due to the lower hydration at low w/b ratios. This indicates that there is still a large quantity of unreacted reactants in the samples. Moreover, although the w/b ratios are different, their chemical compositions are similar.

### 3.8. ATR-FTIR

**Figure 13:**
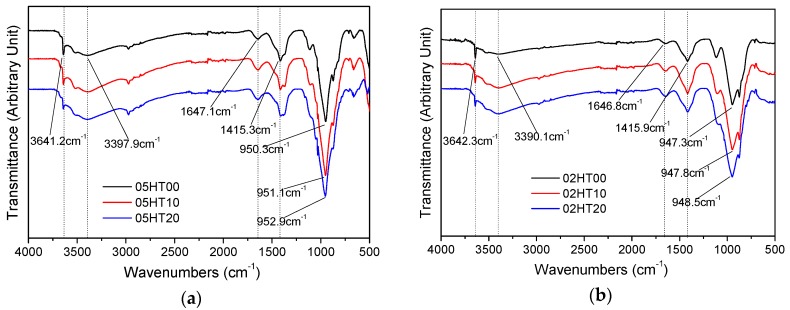
ATR-FTIR spectra for all the samples at the age of 28 days when (**a**) w/b = 0.5 and (**b**) w/b = 0.2.

Figure 13 shows the infrared spectrum of all samples after 28 days, with the spectrum ranging from 500 to 4000 cm^−1^. The characteristic wavenumbers and the associated functional groups of Portland cement are listed in Table 4.

For w/b = 0.5 (Figure 13a) and w/b = 0.2 (Figure 13b), the stretching vibration of Si–O–Si (Si–O–Al) is evident in the 968–971 cm^−1^ range. Moreover, compared with the control, after the addition of the HT powder, the absorption peak of Si–O–Si (Si–O–Al) shifts toward a higher wavenumber. For w/b = 0.5, the 05HT00 wavenumber is 950.3 cm^−1^, the 05HT10 wavenumber is 951.1 cm^−1^, and the 05HT20 wavenumber is 952.9 cm^−1^. For w/b = 0.2, the 02HT00 wavenumber is 947.3 cm^−1^, the 02HT10 wavenumber is 947.8 cm^−1^, and the 02HT20 wavenumber is 948.5 cm^−1^. This indicates that the aluminum phase and silicon phase in the HT powder changes the ratio of n(Ca)/n(Si) in C-S-H and forms more C-S-H gels. This may be the reason for the increase in compressive strength after the addition of the HT powder.

The absorption peak of Ca(OH)_2_ is caused by the stretching vibration of O–H (3641–3644 cm^−1^). It can be clearly seen that the absorption peak of Ca(OH)_2_ decreases with the addition of the HT powder, and the peak intensity is weakened due to the consumption of CH. This phenomenon is also observed in the results of the thermogravimetric and XRD analyses. The bands at 3390–3408 and 1640–1650 cm^−1^ are due to the stretching vibrations and bending vibration of H_2_O, respectively. The bands in the 1400–1500 cm^−1^ range correspond to the asymmetric stretching of CO_3_^2−^.

### 3.9. Mercury Intrusion Porosimetry

**Figure 14:**
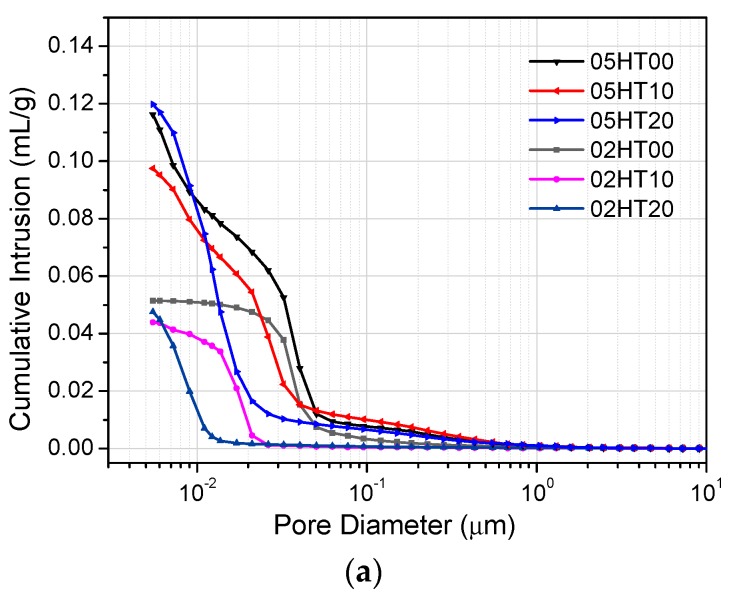

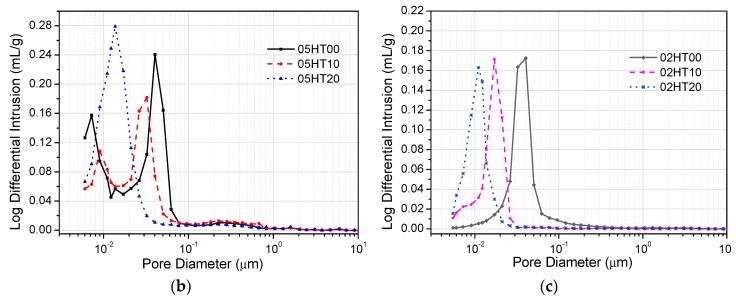
(**a**) Cumulative intrusion volume after 28 days. (**b**) Differential intruded volume when w/b = 0.5. (**c**) Differential intruded volume when w/b = 0.2.

**Figure 15:**
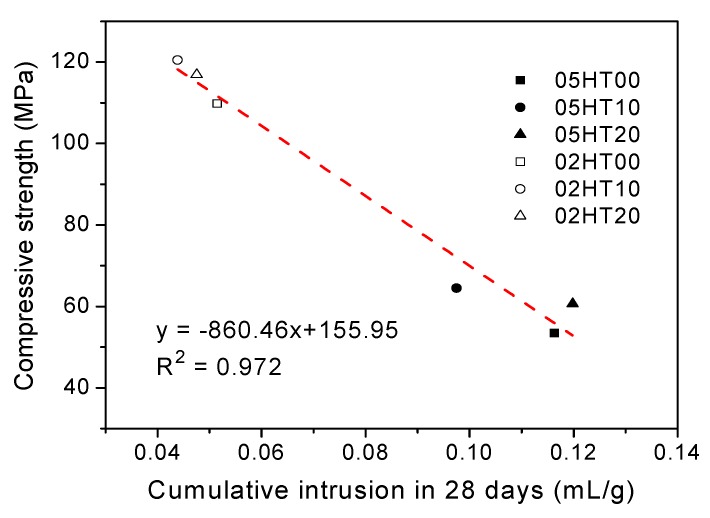
Relationship between compressive strength and cumulative intrusion volume.

Figure 14 shows the cumulative mercury intrusion curves and pore size distribution curves for all pastes after 28 days. It can be clearly seen from Figure 14a that the cumulative pore volume of the paste with a low w/b ratio is significantly lower than that of the one with a high w/b ratio [53,54,55]. Although the cumulative pore volume in the 05HT20 paste is slightly higher than that of the control, the pore size distribution curves of the pastes to which the HT powder was added exhibit pore refinement, as shown in Figure 14b. The addition of the HT powder to the cement results in a paste with a dense microstructure and a finer pore size distribution than the OPC paste. In the analysis results of the infrared spectrum (Section 3.8), changes in the absorption peak of Si–O–Si (Si–O–Al) are also observed. This may be due to the higher degree of polymerization of C-(A)-S-H due to the pozzolanic reaction of the HT powder. This helps the microstructure of the paste to become denser [56]. Figure 15 shows the relationship between the cumulative mercury intrusion volume of the paste and the compressive strength. The correlation coefficient (R^2^) is 0.972. That is, as the cumulative mercury intrusion volume increases, the compressive strength decreases.

### 3.10. Comparison Between Cumulative Heat of Hydration and W_b_

**Figure 16:**
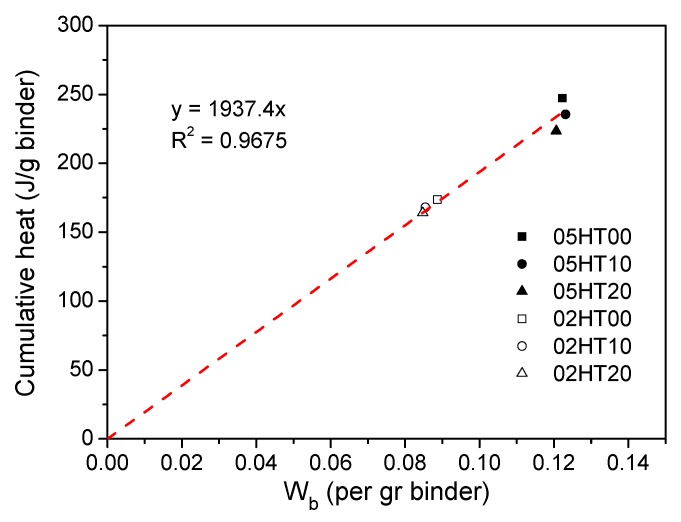
Relationship between cumulative heat and chemically bound water.

Figure 16 shows the relationship between the accumulation of hydration heat and the total amount of W_b_ after the pastes were cured for three days. It can be seen that they have a proportional relationship, i.e., y = 1937.4x (where y is the cumulative heat and x is the W_b_). The R^2^ value is 0.9675. In the results of previous studies [27] and Pane and Hansen’s results [57], the relationship between cumulative hydration heat and W_b_ is also roughly linear, with a slope of 2000.

### 3.11. Comparison Between Compressive Strength and W_b_/W_0_.

**Figure 17:**
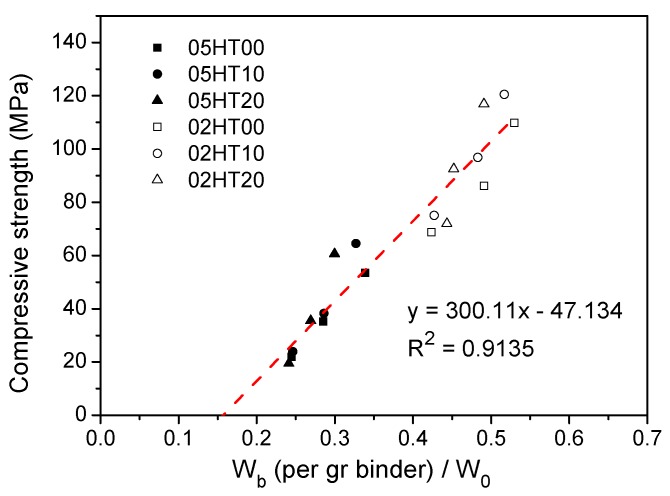
Relations between compressive strength and W_b_/W_0_.

Figure 17 shows the compressive strength as a function of W_b_/W_0_ (where W_b_ is chemically bound water and W_0_ is total water). Linear regression shows that the compressive strength has a good linear relationship with W_b_/W_0_, i.e., y = 300.11x − 47.134. The R^2^ value is 0.9135. Generally, the cementitious material has compressive strength after the final setting. Therefore, the fitted straight line does not pass through the origin, and the strength of the paste rises after a certain amount of W_b_ is generated.

## 4. Further Research Focuses

This article presents the results of a preliminary study. As a new type of complementary cementitious material with great potential in Korea, HT needs to be further researched like other new materials. One of the main research goals is to better understand the relationship of parameters between HT and other mixtures, such as the effect of the particle size of the HT powder, the workability of HT in fresh concrete, the durability of HT-concrete, and the long-term hydration performance. It is also necessary to perform similar analyses in a different set of materials (other cement, calcined clay produced in other conditions) and determine whether HT is suitable for other particular applications, such as coating mortar and repairing mortar. In addition, the degree of the pozzolanic reaction of HT should be detected by selective dissolution method and backscattered electron (BSE) image analysis [58]. Finally, the environmental impact and production costs of HT-concrete industrial production must be further evaluated.

## 5. Conclusions

In this study, the compressive strength, hydration process, and microstructure of calcined Hwangtoh clay-blended cement pastes with a w/b of 0.5 and 0.2 were investigated through a series of experiments. According to the results and discussion, we can derive the following conclusions.

1. Calcined HT can be used to produce concrete. When the replacement ratio of HT powder is 10%, HT-blended cement paste has the highest compressive strength after three, seven, and twenty-eight days. When the replacement rate of HT is 20%, the compressive strength is higher than that of the control paste after curing for twenty-eight days, regardless of the w/b ratio. However, the strength of samples with 20% HT is reduced after three days, regardless of the w/b ratio. Moreover, the HT-blended cement pastes with a w/b ratio of 0.2 show improvements in compressive strength sooner than the pastes with a w/b of 0.5.
2. The HT powder enhances the peak of hydration heat as the amount of HT added increases. Compared with pure cement, HT-containing paste has a reduced cumulative heat, which is normalized per gram of binder. The cumulative hydration heat value at a w/b of 0.5 is higher than at a w/b of 0.2.
3. The Al^3+^ ion concentration in the HT-blended cement paste is higher than that of the OPC in the early stage, thus promoting the formation of ettringite.
4. As the replacement ratio of HT increases, chemically bound water and calcium hydroxide decrease. As the w/b ratio decreases from 0.5 to 0.2, chemically bound water and calcium hydroxide also decrease. In addition, the cumulative heat is directly proportional to the amount of chemically bound water. The compressive strength has a linear relationship with W_b_/W_0_.
5. ATR-FTIR shows that the structure of C-(A)-S-H gels changes in samples with the addition of the HT powder. According to the MIP experimental analysis, the pore structure of HT-blended cement paste is more refined.

## Figures and Tables

**Table 1:** Oxide chemical compositions measured with X-ray fluorescence (XRF) spectroscopy in weight %.

Samples	SiO_2_	Al_2_O_3_	Fe_2_O_3_	CaO	MgO	Na_2_O	TiO_2_	SO_3_	ZnO	P_2_O_5_	K_2_O	LOI ^1^
Cement	21.7	5.77	2.5	62.9	2.4	0.45	0.22	2.34	0.11	0.09	1.03	0.46
Hwangtoh	63.9	24.4	6.13	0.16	0.67	-	0.78	-	-	0.09	3.06	0.83

^1^ Loss on ignition.

**Table 2:** Mix design information in this study.

Mix No.	w/b	Cement		Hwangtoh		Water	Superplasticizer		Flow
		wt.% of Binder	kg/m^3^	wt.% of Binder	kg/m^3^	kg/m^3^	wt.% of Binder	kg/m^3^	(mm)
05HT00	0.5	100	1221.8	0	0	610.9	0	0	255
05HT10	0.5	90	1094.4	10	121.6	608.1	0	0	246
05HT20	0.5	80	968.3	20	242.1	605.2	0	0	231
02HT00	0.2	100	1910.3	0	0	382.1	0.6	11.5	221
02HT10	0.2	90	1696.0	10	188.4	376.9	1.0	18.8	220
02HT20	0.2	80	1487.3	20	371.8	371.8	1.4	26.0	216

**Table 3:** Al^3+^ ion concentration after one day.

Mixtures	100% OPC	90% OPC + 10% HT	80% OPC + 20% HT
Intensity	41286.9	49538.0	61733.5
Al^3+^ ion concentration at one day (μg/L)	2.24	2.69	3.36

**Table 4:** Positions of infrared bands and functional groups in attenuated total reflection Fourier transform infrared spectroscopy (ATR-FTIR) spectra of OPC paste.

Wavenumber (cm^−1^)	Functional Groups	Reference
968–971	Si–O–Si (Si–O–Al), υ_3_	[43,44,45]
1400–1500	C–O (CO_3_^2−^),υ_3_	[25,46,47,48]
1640–1650	H_2_O, υ_2_	[49,50]
3390–3408	H_2_O, υ_1_,υ_3_	[48,51]
3641–3644	Ca(OH)_2_	[25,52]
